# Transformative eating disorder research: qualitative research considerations

**DOI:** 10.1186/s40337-021-00428-2

**Published:** 2021-06-16

**Authors:** Margaret G. Janse van Rensburg

**Affiliations:** Carleton University, Ottawa, Canada

**Keywords:** Qualitative research, emancipatory research, diversity studies, stigma, eating disorders, community-based research

## Abstract

This letter identifies the potential of qualitative eating disorder research to work within a transformative paradigm that naturalizes the state of living with an eating disorder. The number of qualitative research publications with persons living with eating disorders have increased, however, a substantive proportion of this qualitative research follows traditional research paradigms that are built upon the assumption that eating disorders signify a personal deficit. Transformative qualitative eating disorder research has potential to include those living with eating disorders in all stages of the research process to ensure that research leads to the de-stigmatization of eating disorders promoting health, wellbeing, and quality of life for persons living with eating disorders.

## Main text

In recent years there has been an increase in qualitative eating disorder research [1] which explores the experiences of persons living with eating disorders. However, a number of these studies struggle to grasp the importance of conducting qualitative research in natural settings [2, 3]. Much of this qualitative research has also been grounded in traditional research paradigms, and therefore lack critical and emancipatory strategies, including discourse and discursive analyses, which may have transformative implications for bettering the lives of persons living with eating disorders. Qualitative research, as research in natural settings, should also seek to *naturalize* the state of living with an eating disorder, rather than using research to legitimize disparate treatment. This recognizes eating disorders as a natural phenomenon, a valid state of being, and promotes autonomy and self-determination surrounding treatment and recovery.

Historically, eating disorders have been positioned as individualised, being caused by personal deficits [1, 2, 4]. I, while taking part in eating disorder hospital programs and as a participant in an, an rTMS randomized control trial on bulimia, had my own data collected. These research opportunities were hopeful for myself, as they held potential for me to get better. However, they also personalized my issue, rather than acknowledged the structural and societal contributors to my eating disorder development and maintenance [5].

In my work with disabled and autistic^1^ advocates, I have learned approaches to naturalize diversity, promoting inclusivity and acceptance in our society. However, the societal de-stigmatization efforts I encourage for those whom I work with have never extended to me, a woman living with an eating disorder. I am a member of a minority group, an over-researched population, a divergent body and mind. My differences, however, are a cause for concern, something to treat, cure, and eliminate. Yes, the health risks of eating disorders are real, however, I believe that traditional research which promotes the idea of individual deficit does not lead to transformative personal and societal outcomes bettering the lives of persons living with eating disorders.

Inspired by academics in the field of Critical Autism Studies [6], where autistic authors have begun to question the role of research in perpetuating stigma and power imbalances between autistic persons and a perceived *normal* [7]. I have begun to question the role of the dominant eating disorder research in stigmatizing my state of being. Informed by principles of community based participatory research [9], action research [10], and post-colonial theory [8], could qualitative eating disorder research occurring with, rather than on, persons living with eating disorders, lead to transformative outcomes?

I encourage future eating disorder researchers to involve stakeholders in their research [9, 10], to recognize those who are researched as experts on their own lived experience [7] and to critically reflect on how research decisions may have the potential to perpetuate the stigmatization of the persons they aim to assist. Ways to address this are outlined in Table 1.

**Table 1:** Differentiating Traditional and Transformative Eating Disorder Research

	Traditional Eating Disorder Research	Transformative Eating Disorder Research
Research topics and questions	The *cause* and *cure* of eating disorders; practitioner curiosity.	Research driven by community wants and needs.
Research methods	Participant is unaware of what is being measured and why it is being measured.	Open and transparent process where the participant can contribute to how the research proceeds.
Analysis	Researchers are in control of the data used and can make assumptions about what participants wants and needs are. No shared ownership of data.	Participant is part of the research team, is involved and aware of assumptions. Member-checking and other strategies are used to ensure that the analysis leads to destigmatizing attitudes towards eating disorders.
Findings	Seeking to *normalize* persons living with eating disorders: make them “better.”	Seeking to *naturalize* persons living with eating disorders: destigmatizing coping, allowing autonomy and self-determination, and promoting health and well-being with or without eating disorder “recovery.”
Outcomes	Personal responsibility of the individual to get “better.”	Advocating with persons living with eating disorders for better societal outcomes.

## Data Availability

N/A

## Footnotes

1 I use the term disabled and autistic in solidarity with my disabled friends and allies who prefer identity-first language, and in line with field of Critical Autism Studies [6–8].
